# Peritoneal Dialysis Catheter Placement in Children: Initial Experience with a “2+1”-Port Laparoscopic-Assisted Technique

**DOI:** 10.3390/medicina59050961

**Published:** 2023-05-16

**Authors:** Vlad-Laurentiu David, Elisa Mussuto, Ramona-Florina Stroescu, Mihai Gafencu, Eugen-Sorin Boia

**Affiliations:** 1 Department of Pediatric Surgery and Orthopedics, “Victor Babes” University of Medicine and Pharmacy Timisoara, 300041 Timisoara, Romania; 2 Department of Pediatric Surgery, Fondazione I.R.C.C.S. Policlinico San Matteo, Via Forlanini, 16, 27100 Pavia, PV, Italy; 3 Departments of Pediatrics, “Victor Babes” University of Medicine and Pharmacy Timisoara, 2 Eftimie Murgu, 300041 Timisoara, Romania

**Keywords:** peritoneal dialysis, catheter, laparoscopy, pediatric, child, omentectomy

## Abstract

The placement of a peritoneal dialysis catheter (PDC) is currently a common procedure in pediatric surgeon practice, and the search for the ultimate technique never stops. The purpose of this study is to evaluate our experience with the laparoscopic PDC placement approach, performing a “2+1” (“two plus one”) technique, where the “+1” trocar is placed in an oblique manner, pointing toward the Douglas pouch when passing through the abdominal wall. This tunnel is further used to place and maintain the proper position of the PDC. Materials and methods: We assessed a cohort of five children who underwent laparoscopic-assisted PDC placement between 2018 and 2022. Results: This procedure is a simple, relatively quick, and safe technique for PDC placement. Furthermore, in our experience, concomitant omentectomy is necessary to reduce the risk of catheter obstruction and migration due to omental wrapping. Conclusions: The laparoscopic approach allows for improved visualization and more accurate placement of a catheter inside the abdominal cavity. Concomitant omental excision is necessary to prevent PDC malfunction and migration.

## 1. Introduction

The use of peritoneal dialysis to treat renal failure in pediatric population was first reported in the 1940s [1]. Nowadays, peritoneal dialysis is frequently used as renal replacement therapy in pediatric patients with either chronic or acute renal failure [2,3,4].

Peritoneal dialysis provides adequate clearance and correction of metabolic imbalances as well as ultrafiltration and fluid delivery, and it can be easily used to treat children of all ages and sizes, ranging from newborns to adolescents [3,4,5]. Peritoneal dialysis has certain advantages over other renal replacement strategies; it is easier to perform than hemodialysis, less invasive, physiological, less pro-inflammatory, does not require vascular access, and may be performed as an outpatient procedure [2,3,4,5,6]. Moreover, the structure of the peritoneal membrane permits clearance of higher molecular weight substances thanks to its large pores [3]. Peritoneal dialysis is particularly advantageous in the pediatric population, as children have a larger peritoneal surface area (unit per weight) compared to adults, allowing for more effective fluid clearance [3]. Some other advantages of peritoneal dialysis are its lower cost compared with other types of replacement therapy and the simplicity of the procedure [2,3].

While percutaneous insertion using the Seldinger technique or interventional radiological techniques may be an option for PDC placement in certain cases, the surgical approach—inside the operating theater with the patient under general anesthesia—is performed more often, particularly in children [5,6,7]. The percutaneous approach has a higher risk for morbidity and peri-procedural complications and is not suitable for patients with previous abdominal surgery or for those with end-stage renal disease. In these particular patients, due to higher risks for complications, the open technique or the laparoscopic one are the procedures of choice [6,7]. In children, considering the size of the abdominal cavity and the low compliance of the patients, the percutaneous techniques are even less feasible, and the PDCs are placed almost exclusively by surgical means [5].

Surgical peritoneal dialysis catheter (PDC) placement was developed as a successful RRT method in the first half of the last century [8,9,10,11,12]. During the early 1990s, descriptions of laparoscopic placement of PDC began appearing in the literature. Considering the irrefutable advantages of minimally invasive surgery, many surgeons have now adopted the laparoscopic approach for PDC insertion [13]. However, no golden standard surgical technique for PDC insertion has been declared, and many variants independently coexist [14,15,16].

Moreover, there is still debate among specialists as to whether the laparoscopic approach has clear advantages over open techniques [14]. Although open surgery has the advantages of simplicity, low cost, and no need for special equipment, it is associated with higher morbidity and a higher risk of post-procedural complications [6]. A laparoscopic approach demonstrates better accuracy in PDC placement and less morbidity due to less mechanical trauma [6,7]. On the other hand, laparoscopy requires dedicated equipment and trained personnel, and thus higher costs [6,7]. Many different factors may influence the procedure of PDC placement and the lifespan of the catheter (age, weight, preexisting cardiac, pulmonary pathologies, or prior abdominal surgery) [13,14,15,16,17].

The aim of this study is to present our initial experience with the laparoscopic surgical technique for PDC placement in a series of five consecutive cases.

## 2. Materials and Methods

### 2.1. The Patients

Results from a population of five children (*n* = 5) who underwent laparoscopic PDC placement at the Department of Pediatric Surgery of the “Louis Turcanu” Emergency Children’s Hospital Timisoara over a four-year period, from 2018 to 2022, are reported. All the patients were diagnosed with grade IV and V chronic renal disease. The only inclusion criterion was laparoscopically-assisted insertion of the PDC. The cohort comprised 1 female (20%) and 4 males (80%). The median age was 12 (6–12) years. The median weight was calculated as 25 (17–55) kg. Perioperative and demographic data are shown in Table 1. Patient and perioperative data were determined for each surgery by evaluating the total operative time, the operative time for catheter placement, and the size of the catheter, as illustrated in Table 1.

### 2.2. Operative Technique

**Figure 1:**
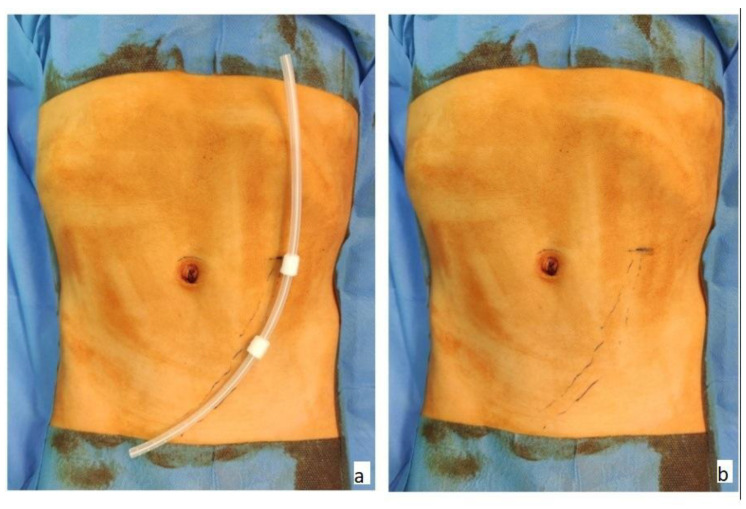
Marking the desired position of the catheter and the entry point.

General anesthesia with orotracheal intubation was administered to all patients. The patient was placed in the supine position on the operating table and, prior to surgery, the skin was marked to indicate the point of PDC insertion in addition to the presumptive trajectory through the abdominal wall and inside the peritoneal cavity (Figure 1a,b).

**Figure 2:**
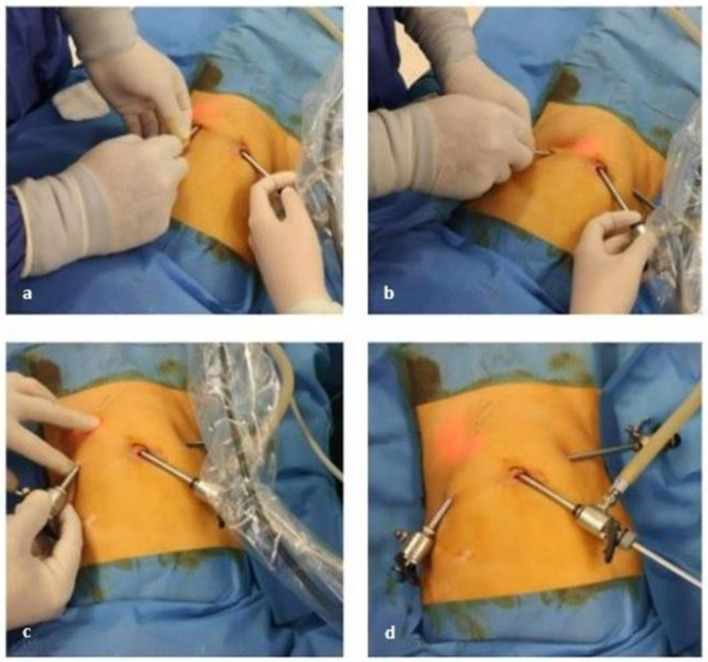
Position of the ports and insertion of the third trocar. The third port was inserted in an oblique orientation, passes through the abdominal wall, and enters the peritoneal cavity, following the external drawings and guided by the light source.

The surgeon stayed on the patient’s side on which the catheter was to be inserted. The monitor was placed across the table, opposite to the surgeon. The assistant stood next to the surgeon and the scrub nurse stood at the opposite side of the table, next to the monitor. We used a standard three 5 mm port approach. The first cannula was placed using an open, supra-umbilical approach, and the pneumoperitoneum was created. The second port was placed in the right iliac region. The third port was then placed in the left flank to later be used as the insertion site for the Tenckhoff catheter (Figure 2a). Its position is in accordance with the previously marked points and can vary according to the size of the catheter, the age, weight, and height of the patient. Despite various types of catheters being available on the market (straight or arcuate, single or double or disc-shaped inner cuff with silicone ball), we preferred the straight double cuff type catheters. Even though the single cuff catheters are easy to remove, we preferred the double cuff ones in order to increase the fixation of the latter and to avoid complications related to its position in between the abdominal wall layers [1].

The third port was inserted in an oblique fashion inside the abdominal wall, pointing towards the Douglas pouch in order to create a tunnel to accommodate the catheter. We used the trans-luminescence of the optic pointing against the abdominal wall as landmark to ensure the right direction of the tunnel (Figure 2a,b). The insertion angle is usually between 10 and 20 degrees, with variations depending on the anatomical characteristics of each patient (i.e., subcutaneous and muscular layer width). By modifying the insertion angle, the trocar passed a few cm in the subcutaneous tissue, then through the muscular layer, and finally a few cm between the muscle and the peritoneum (Figure 2a–d).

**Figure 3:**
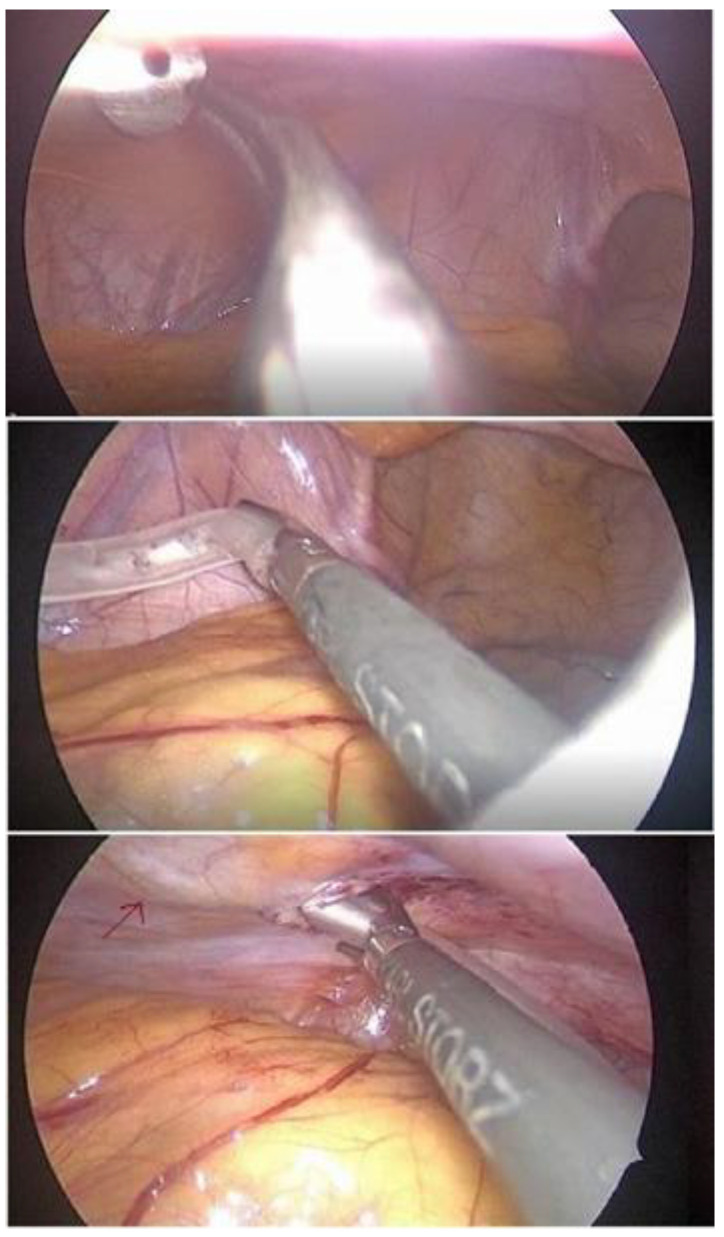
The tip of the catheter was grasped and pulled down to the Douglas pouch, taking care to place the distal cuff in the proper position (red arrow).

The trocar from the third port was removed and the catheter was inserted through the port until its distal tip passed into the abdomen. The distal tip of the catheter was then grasped and held in place by retracting forceps while the port was retracted, sliding over the catheter. Care must be taken to block the evacuation of the pneumoperitoneum through the catheter. When the third port was entirely removed, the catheter was gently retracted through the tunnel into the peritoneal cavity until the tip reached the Douglas pouch. Care must simultaneously be taken to place the distal cuff in the proper position (Figure 3).

**Figure 4:**
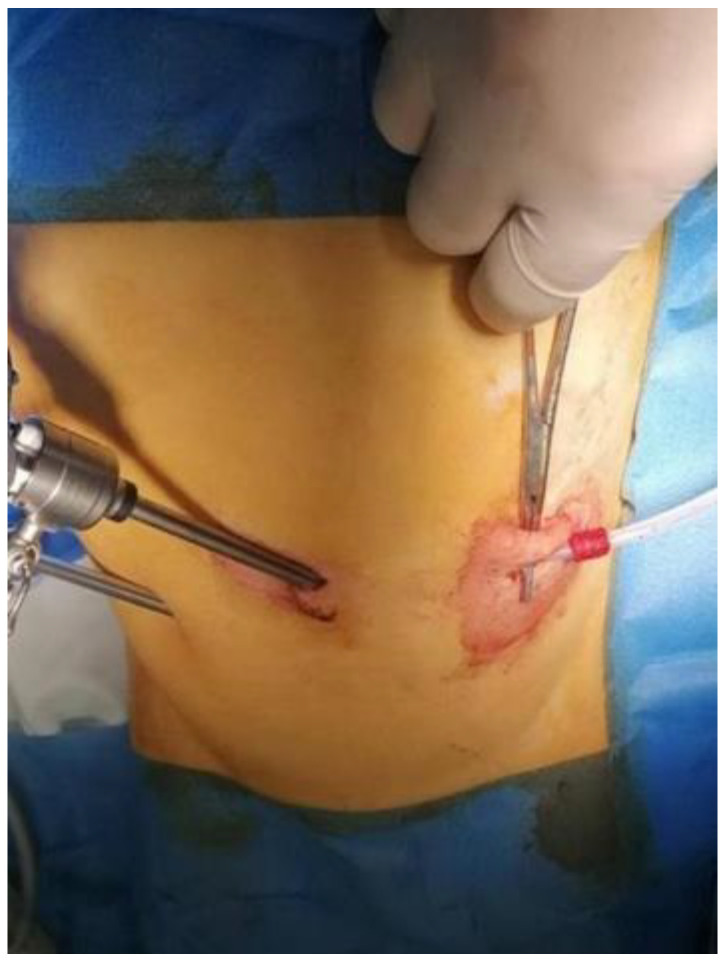
The proximal cuff was placed in the subcutaneous tissue and the catheter was secured to the skin.

**Figure 5:**
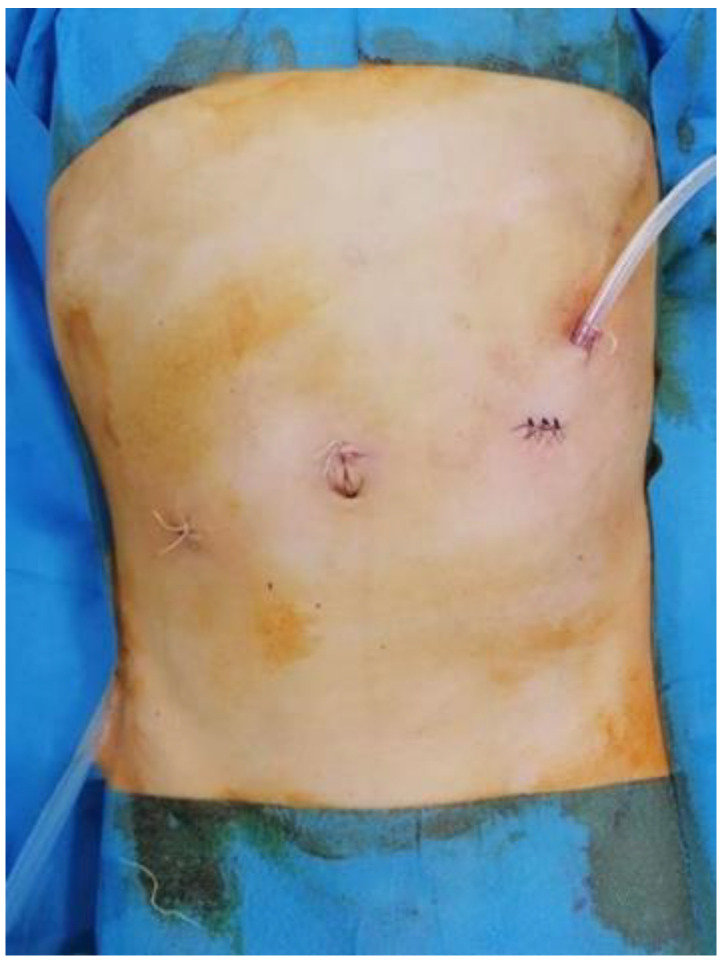
The final aspect.

The proximal cuff was placed in the subcutaneous tissue and the skin was closed around the catheter. If required, a second incision through which the catheter is externalized can be placed cranially to the first one (Figure 4). PDC was then tested by the infusion of 20 mL of saline solution and aspiration. A non-resorbable stitch was placed around the catheter for external security. The pneumoperitoneum was evacuated, and the abdominal incisions were closed using simple absorbable stitches (Figure 5).

The above-presented surgical procedure was slightly different for the last two cases, for which concomitant omentectomy was performed. The first step of the surgical procedure is the omentectomy and the setup, accordingly [18]. The surgeon stood on the left side of the patient facing the cranial and the monitor was on the opposite side facing the caudal. The first two ports were placed as described above—the optic one in the supra-umbilical area and the second in the right iliac region. The third port was first inserted perpendicular to the abdominal wall in order to perform the omentectomy. The omentectomy was carried out using a combination of bipolar, monopolar diathermy, and scissors. The resected omentum was removed through the port on the left flank. After the omentectomy was completed, the setup of the surgical table was changed and the third port was repositioned, as described above, for the PDC placement.

## 3. Results

The median total operative time was 60 (47–90) minutes. The median operative time for PDC placement was 15 (13–20) minutes. All the procedures were completed laparoscopically. No accidental iatrogenic injuries to abdominal organs during the surgery were reported, nor were other peri-procedural complications. The immediate course of the patient was favorable in all five patients, and peritoneal dialysis could be initiated a few days after surgery. Complications occurred in two cases (patients 2 and 3).

In patient 2, the PDC became inefficient approximately 2 weeks after surgery. Abdominal ultrasound revealed that the tip of the catheter was no longer in the Douglas pouch. Using a laparoscopic approach, we performed omentectomy and replaced the tip of the PDC in the Douglas pouch. The omentum below the transverse colon was removed and taken out through one of the 5 mm working ports. The further course of the patient was uneventful.

**Figure 6:**
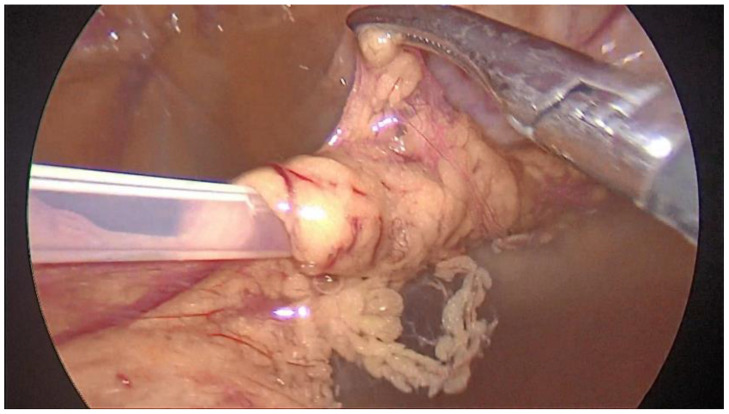
Peritoneal dialysis catheter wrapped by omentum and blocked in patient 3.

In patient 3, the catheter became ineffective approximately 4 weeks after the initial surgery. Fluid could be inserted, but evacuation was blocked. An abdominal ultrasound revealed the occurrence of catheter migration. We performed a second laparoscopy and found that the tip of the PDC did not reach the Douglas pouch and was located near the dome of the urinary bladder. Moreover, it was partially covered by the omentum (Figure 6). We proceeded to further advance the catheter toward the Douglas pouch until the tip was placed in the correct position and performed concomitant omentectomy. The subsequent course of the patient was also uneventful.

Omentectomy alone was enough to ensure a good functioning of the device without any further mechanical complications. In fact, for the rest of the patients we performed omentectomy concomitantly with the catheter insertion, without any mechanical or functional complications. No exit site infection or other abdominal wall complications were reported during the surgical follow-up of these patients.

## 4. Discussion

Many laparoscopic-assisted techniques for PDC placement have been described, and their validity and safety upon performance has been demonstrated in all cases [18,19,20]. Since 1993, the Mid-European Pediatric Peritoneal Dialysis Study Group (MEPPS) has evaluated more than 200 children, accumulating epidemiological data regarding the PDC placement in the pediatric population [21]. Their conclusion was that technique survival rates of peritoneal dialysis in children are similar to those reported for adults, despite young children being at increased risk for peritonitis [21].

In relation to the advantages of the laparoscopic approach versus the open placement of PDC, opinions among specialists are varied. Some meta-analysis and prospective studies revealed no superiority of laparoscopic techniques over the open approach [16,22,23]. A 10-year review of acute peritoneal dialysis catheter placement by Maria Stack et al. showed no significant differences in complication rates between laparoscopic and open surgical approaches. However, an increased leakage with laparoscopic procedures has been reported [24]. Moreover, on the basis of being less time-consuming and demanding of technical resources, the open approach is considered cost-effective and superior [22]. However, the advantages of minimally invasive approaches, regardless of the surgical procedure performed, are well known. Perhaps one of the most important features of laparoscopy is that it allows direct visualization of the peritoneal cavity. A surgeon can thus properly assess the interior of the abdominal cavity and perform lysis of the adhesions, when necessary, and more accurately place the PDC [17]. Moreover, laparoscopic placement of peritoneal dialysis catheter is feasible in children of all age groups with functional results similar to those obtained with the open approach [25]. A recent meta-analysis confirmed that laparoscopic placement of peritoneal catheters is effective and associated with fewer postoperative complications [26]. Where possible, the current guidelines do recommend laparoscopy as the first choice for PDC placement in both adult and child patients that require renal replacement therapy [17,20].

There are a few key points related to our surgical technique. First, prior to the creation of the pneumoperitoneum and starting the surgical procedure itself, we determined and marked the ideal trajectory of the PDC, point of incision, and the peritoneal entry point. This is a very useful step because, once the peritoneum is created, these points will move from their normal location. The second key point is the way the third port is inserted. We deliberately inserted this port in an oblique fashion through the abdominal wall. The placement of the port in this fashion allows for the creation of a longer tunnel inside the abdominal wall, securing the correct positioning of both the deep and superficial cuffs. This fashion of the catheter and the presence of the cuffs help to fix the catheter and to minimize the risk of infection, blocking the migration of microorganisms into the peritoneal cavity by the peri-catheter route [27]. Even if many studies demonstrated the increased risk of infection if the catheter tip exit site is placed upward, thus preferring a lateral or downward direction of the catheter exit point, many centers use double-cuff curled Tenckhoff catheter with an upward pointing exit site [1,20]. As a matter of fact, we did not have any exit site infection besides the upward position.

The catheter was then introduced through the port, which permits a clear and easy passage. After retraction of the port, the abdominal wall tissue will contract over the body of the catheter and ensure a good seal of the peritoneal cavity. The use of the third port as a guide allows for the creation of a clear pathway in between the abdominal wall layers and the peritoneal cavity, making insertion simple and thus leading to the fixation of the PDC. Trauma to the abdominal wall is minimal; there is no cutting into the muscle or aponeurosis for which healing is required. The International Society for Peritoneal Dialysis (ISPD) recommends a break-in period of 10–15 days between the insertion of the PDC and the initiation of the peritoneal dialysis in order to avoid peri-catheter leaks [28]. On the other hand, some authors have reported an increased risk for catheter failure if dialysis was performed within three days of catheter placement, recommending, if possible, to wait at least three days after surgery to start the therapy in order to allow for healing of the wounds [29]. Thus, the risk of complications and catheter failure is significantly reduced, and the lifespan of the device is increased [29]. A recent 2021 study published by Bawazir et al. reported that, even for open placement with associated omentectomy, the early start of the peritoneal dialysis is possible [30].

In the case of laparoscopic PDC placement, after retraction of the port, the structures of the abdominal wall will retract more tightly over the catheter, preventing further fluid leakage. This is particularly important because dialysis may be initiated immediately after surgery using this technique, in contrast to the open technique, where the clinician has to wait—sometimes up to 14 days—for the abdominal wound to heal. Indeed, several studies have confirmed that laparoscopy is superior to open surgery for preventing fluid leakage after PDC placement [20,31,32].

Unfortunately, complications in peritoneal dialysis are common, particularly in pediatric patients [2]. The most common complications reported are infection, fluid leak, obstruction of the catheter, and catheter migration, as well as bowel perforation and occlusion [2,32,33,34]. Even if we did not see any, peritonitis is reported as the most common complication in many studies affecting the normal functioning of the catheter, requiring revision of the system [35,36]. Infectious complications can be limited by adequate antiseptic measures of the catheter placement site and during fluid exchange. However, infectious causes of access revision show a decreased risk of technique failure when compared to access revision due to mechanical causes. Mechanical malfunction, peritonitis, exit site infection, and leakage represent the main reasons for access revision. The need for access revision increased the risk of technique failure. In most cases, catheter obstruction and migration occur due to incorrect placement or due to omental wrapping of the tip of the catheter [20,32,33].

One of the major advantages of laparoscopy over the open approach is that the trauma over the abdominal wall is less extensive [13]. The tunnel created by the laparoscopic trocar, when passing the abdominal wall, will close immediately after the trocar is removed. This happens because the tissue is stretched, not cut, as in open techniques, and will retract back when the trocar is removed. This aspect is particularly important in PDC placement when there is a need to seal the peritoneal cavity in order to avoid fluid leakage. Dialysate fluid leakage may occur in up to 22% of the cases, even with the laparoscopic approach [3,37]. Fortunately, we did not have any pericatheter leakage in our series, highlighting the advantages of the laparoscopic approach.

We did experience such complications in two of our first three cases. We had to re-operate on these patients and, under laparoscopic visualization, the cause of obstruction was identified as omental wrapping of the PDC tip. We performed omentectomy on these patients and then decided to perform omentectomy as a routine step in the procedure for subsequent cases. Lee et al. used laparoscopic omentectomy and catheter fixation to salvage non-functioning peritoneal dialysis catheters [35]. They showed that laparoscopic omentectomy with catheter fixation is a useful method to restore PDC in which there was prior obstruction by the omentum [35]. A few other studies have confirmed that concomitant omentectomy is safe and reduces the incidence of catheter obstruction during short- and long-term use and significantly improves the overall PDC survival [38,39,40,41,42,43]. Omentopexy or omental folding have been described as alternative options to an omentectomy performed in adult patients [44,45]. However, it might be dangerous to create intra-peritoneal fix points, and these might lead to mechanical complications in children. Moreover, with careful dissection and adequate hemostatic measures, omentectomy can be performed bloodlessly. We did not experience excessive intraoperative or postoperative bleeding in our series of concomitant omentectomy and PDC placement. Finally, the further course was favorable in all five patients despite the complications in the second and third cases. Since we reached the goal of the procedure in all five cases, meaning a working PDC, we considered the result as favorable for each of the five cases.

The only criticism of laparoscopic PDC placement is the prolongation of the operative time. Some of the published studies have shown a significantly longer operative time with the laparoscopic technique instead of the conventional open one [24]. The mean operative time in our study, 55.5 ± 6.34 min, is similar to those of previous reports [46]. However, concomitant omentectomy has a significant impact on operative time, whereas the time for catheter placement is reasonably low (14.75 ± 1.48 min). We believe this is a small price to pay when weighed against the advantages of allowing the surgeon to be able to insert the catheter based on direct visualization to ensure the correct positioning of the device at the end of the procedure, thus reducing the risk for malfunctioning and catheter migration.

The limitation of this study is its small number of cases. This is mainly because there are not many cases with the indication for peritoneal dialysis in children. Generally speaking, there is a paucity of published experience in children regarding PDC placement and access revisions, making it hard to create a golden standard. In addition, due to the small sample size of the published studies, many of them show conflicting results. That is why a continuous improvement in surgical techniques, PDC design, and, most of all, the creation of a large database are essential to ameliorate the procedure, to compare adequacy between different techniques, to evaluate the impact of the procedures on outcomes, to understand the pros and cons of different approaches, and to better assess the morbidity rate [3,37,47]. Future studies following this initial investigation are planned to further assess this technique.

## 5. Conclusions

Laparoscopic-assisted PDC insertion is safe and effective. The laparoscopic approach allows for improved visualization and more accurate placement of the catheter inside the abdominal cavity, reducing post-procedural risk for PDC malposition. Concomitant omental excision is necessary to prevent PDC malfunction and migration. The laparoscopic technique is a clear, bloodless procedure with minimal trauma over the abdominal wall and allows a good cosmesis of the abdominal wall. The laparoscopic approach also permits a faster recovery and thus early inception of peritoneal dialysis.

## Figures and Tables

**Table 1:** The patients.

Patient	Age	Sex	Weight (kg)	Total Operative Time (min)	PDC Placement (min)
1	6	F	17	60	15
2	16	M	45	63	17
3	9	M	20	52	14
4	17	M	55	47	13
5	12	M	25	90	20

## Data Availability

The data used and/or analyzed during the present study are available from the corresponding author on reasonable request.

## References

[1] Emir H., Bax K.M.A., Georgenson K.E., Rothenberg S.S., Valla J.S., Yeung C.K. (2008). Endoscopic Surgery for Peritoneal Dialysis Catheter in Children. Endoscopic Surgery in Infants and Children.

[2] Rasmussen S.K. (2022). An overview of pediatric peritoneal dialysis and renal replacement therapy in infants: A review for the general pediatric surgeon. Semin. Pediatr. Surg..

[3] Vasudevan A., Phadke K., Yap H.K. (2017). Peritoneal dialysis for the management of pediatric patients with acute kidney injury. Pediatr Nephrol..

[4] Fraser N., Hussain F.K., Connell R., Shenoy M.U. (2015). Chronic peritoneal dialysis in children. Int. J. Nephrol. Renov. Dis..

[5] Nourse P., Cullis B., Finkelstein F., Numanoglu A., Warady B., Antwi S., McCulloch M. (2021). ISPD guidelines for peritoneal dialysis in acute kidney injury: 2020 Update (paediatrics). Perit. Dial. Int..

[6] Zhang D., Peng Y., Zheng T., Liu H., Wu J., Li Z., Xu Y., Hu X., Chen G., Hou H. (2020). An analysis of the “Half-Perc” versus open surgical placement method for a peritoneal dialysis catheter: A non-inferiority cohort study. BMC Nephrol..

[7] Zhu W., Jiang C., Zheng X., Zhang M., Guo H., Yan X. (2015). The placement of peritoneal dialysis catheters: A prospective randomized comparison of open surgery versus “Mini-Perc” technique. Int. Urol. Nephrol..

[8] Sanderson K.R., Harshman L.A. (2020). Renal replacement therapies for infants and children in the ICU. Curr. Opin. Pediatr..

[9] Pyper R.A. (1948). Peritoneal Dialysis. Ulster Med. J..

[10] Tenckhoff H., Schechter H. (1968). A bacteriologically safe peritoneal access device. Trans. Am. Soc. Artif. Intern. Organs.

[11] Devine H., Oreopoulos D.G., Izatt S., Mathews R., DeVeber G.A. (1975). The permanent Tenckhoff catheter for chronic peritoneal dialysis. Can. Med. Assoc. J..

[12] Tenckhoff H., Curtis F.K. (1970). Experience with maintenance peritoneal dialysis in the home. Trans. Am. Soc. Artif. Intern. Organs.

[13] Jha V., Abrahams A.C., Al-Hwiesh A., Brown E.A., Cullis B., Dor F.J.M.F., Mendu M., Ponce D., Divino-Filho J.C. (2022). Peritoneal catheter insertion: Combating barriers through policy change. Clin. Kidney J..

[14] Haskins I.N., Schreiber M., Prabhu A.S., Krpata D.M., Perez A.J., Tastaldi L., Tu C., Rosen M.J., Rosenblatt S. (2017). Peritoneal dialysis catheter placement as a mode of renal replacement therapy: Long-term results from a tertiary academic institution. Surgery.

[15] Damle R.N., Munoz-Abraham S., Osei H., Greenspon J.Y., Villalona G.A. (2021). Pediatric Peritoneal Dialysis Catheter Placement: An Anonymous Survey of APSA Members. J. Surg. Res..

[16] Bagul A., Thiyagarajan U.M., Mamode N. (2014). Laparoscopic peritoneal dialysis catheter (PDC) insertion: Does it really make a difference?. J. Nephrol..

[17] Crabtree J.H., Shrestha B.M., Chow K.M., Figueired A.E., Povlsen J.V., Wilkie M., Abdel-Aal A., Cullis B., Goh B.L., Briggs V.R. (2019). Creating and Maintaining Optimal Peritoneal Dialysis Access in the Adult Patient: 2019 Update. Perit. Dial. Int..

[18] Lee J.H., Kim J., Alkatout I., Mettler L. (2018). Surgical Techniques of Laparoscopic Omentectomy. Hysterectomy.

[19] Bustangi N., Khirallah M.G. (2020). Laparoscopic two-port pre-tied peritoneal dialysis catheter insertion in children: A simple modification. Ann. Pediatr. Surg..

[20] Haggerty S., Roth S., Walsh D., Stefanidis D., Price R., Fanelli R.D., Penner T., Richardson W., SAGES Guidelines Committee (2014). Guidelines for laparoscopic peritoneal dialysis access surgery. Surg. Endosc..

[21] Schaefer F., Klaus G., Müller-Wiefel D.E., Mehls O. (1999). Current practice of peritoneal dialysis in children: Results of a longitudinal survey. Mid European Pediatric Peritoneal Dialysis Study Group (MEPPS). Perit. Dial. Int..

[22] Blitzkow A.C.B., Biagini G., Sabbag C.A., Buffara-Junior V.A. (2022). Laparoscopic peritoneal dialysis catheter placement with rectus sheath tunneling: A one-port simplified technique. Arq. Bras. Cir. Dig..

[23] Jwo S.C., Chen K.S., Lee C.C., Chen H.Y. (2010). Prospective randomized study for comparison of open surgery with laparoscopic-assisted placement of Tenckhoff peritoneal dialysis catheter—A single center experience and literature review. J. Surg. Res..

[24] Stack M., Price N., Ronaldson J., Prestidge C., Wong W., Kara T. (2016). Laparoscopic versus open peritoneal dialysis catheter insertion for the management of pediatric acute kidney injury. Pediatr. Nephrol..

[25] Kim H.S., Jung S.M., Lee S.K., Seo J.M. (2011). Comparison of the Laparoscopic and Open Peritoneal Dialysis Catheter Insertion in Children. J. Korean Assoc. Pediatr. Surg..

[26] Abdijalil G., Shuijuan S. (2022). Laparoscopic versus Open-surgery Catheter Placement in Peritoneal Dialysis Patients: A Meta-analysis of Outcomes. Indian J. Nephrol..

[27] Xie H., Zhang W., Cheng J., He Q. (2012). Laparoscopic versus open catheter placement in peritoneal dialysis patients: A systematic review and meta-analysis. BMC Nephrol..

[28] Warady B.A., Bakkaloglu S., Newland J., Cantwell M., Verrina E., Neu A., Chadha V., Yap H.K., Schaefer F. (2012). Consensus guidelines for the prevention and treatment of catheter-related infections and peritonitis in pediatric patients receiving peritoneal dialysis: 2012 update. Perit. Dial. Int..

[29] Imani P.D., Carpenter J.L., Bell C.S., Brandt M.L., Braun M.C., Swartz S.J. (2018). Peritoneal dialysis catheter outcomes in infants initiating peritoneal dialysis for end-stage renal disease. BMC Nephrol..

[30] Bawazir O., Bawazir R. (2021). Surgical Outcomes of Pediatric Peritoneal Dialysis Catheter Function in a Referral Center. Saudi J. Kidney Dis. Transplant..

[31] Hagen S.M., Lafranca J.A., Steyerberg E.W., Ĳzermans J.N., Dor F.J. (2013). Laparoscopic versus open peritoneal dialysis catheter insertion: A meta-analysis. PLoS ONE.

[32] Lemoine C., Keswani M., Superina R. (2019). Factors associated with early peritoneal dialysis catheter malfunction. J. Pediatr. Surg..

[33] Cribbs R.K., Greenbaum L.A., Heiss K.F. (2010). Risk factors for early peritoneal dialysis catheter failure in children. J. Pediatr. Surg..

[34] Lima M., Di Salvo N., Marchi G., Catania V.D., Libri M., Gargano T. (2020). Peritoneal dialysis catheters in pediatric patients: 10 years of experience in a single centre. Pediatr. Med. Chir..

[35] Lee M., Donovan J.F. (2002). Laparoscopic omentectomy for salvage of peritoneal dialysis catheters. J. Endourol..

[36] Borazan A., Comert M., Ucan B.H., Comert F.B., Sert M., Sekitmez N., Cesur A. (2006). The comparison in terms of early complications of a new technique and percutaneous method for the placement of CAPD catheters. Ren. Fail..

[37] Nikibakhsh A.A., Mahmoodzadeh H., Vali M., Enashaei A., Asem A., Yekta Z. (2013). Outcome of immediate use of the permanent peritoneal dialysis catheter in children with acute and chronic renal failure. Iran. J. Pediatr..

[38] Frehat M.Q.F., Al-Salaita G.M., Al-Bderat J.T., Alhadidi A.M., Mohammad S.A., Shaaban A.M., Al Mardini R. (2020). Chronic peritoneal dialysis in children: A single-centre experience in Jordan. Sudan. J. Paediatr..

[39] Nicholson M.L., Burton P.R., Donnelly P.K., Veitch P.S., Walls J. (1991). The role of omentectomy in continuous ambulatory peritoneal dialysis. Perit. Dial. Int..

[40] Wong Y.S., Pang K.K.Y., Ma A.L.T., Tong P.C., Tam Y.H. (2020). A standardized technique of laparoscopic placement of peritoneal dialysis catheter with omentectomy and closure of patent processus vaginalis: A 3-in-1 minimally invasive surgical approach in children. J. Pediatr. Surg..

[41] Reissman P., Shiloni E., Kluger Y., Berlatzky Y. (1989). Routine omentectomy during Tenckhoff catheter insertion for peritoneal dialysis. Harefuah.

[42] Kim J.K., Lolas M., Keefe D.T., Rickard M., Yadav P., Ming J.M., Milford K., Koyle M.A., Lorenzo A.J., Chua M.E. (2022). Omental Procedures during Peritoneal Dialysis Insertion: A Systematic Review and Meta-Analysis. World J. Surg..

[43] Baksi A., Asuri K., Vuthaluru S., Yadav R.K., Prajapati O.P., Bansal V.K., Kumar S., Mahajan S., Bhowmik D., Bagga A. (2022). Does Laparoscopic Omentectomy Reduce CAPD Catheter Malfunction: A Three-arm Pilot Randomized Trial. Indian J. Nephrol..

[44] Crabtree J.H., Fishman A. (2003). Selective performance of prophylactic omentopexy during laparoscopic implantation of peritoneal dialysis catheters. Surg. Laparosc. Endosc. Percutaneous Tech..

[45] Goh Y.H. (2008). Omental folding: A novel laparoscopic technique for salvaging peritoneal dialysis catheters. Perit. Dial. Int..

[46] Copeland D.R., Blaszak R.T., Tolleson J.S., Saad D.F., Jackson R.J., Smith S.D., Kokoska E.R. (2008). Laparoscopic Tenckhoff catheter placement in children using a securing suture in the pelvis: Comparison to the open approach. J. Pediatr. Surg..

[47] Borzych-Duzalka D., Aki T.F., Azocar M., White C., Harvey E., Mir S., Adragna M., Serdaroglu E., Sinha R., Samaille C. (2017). International Pediatric Peritoneal Dialysis Network (IPPN) Registry. Peritoneal Dialysis Access Revision in Children: Causes, Interventions, and Outcomes. Clin. J. Am. Soc. Nephrol..

